# Insertion/Deletion Within the KDM6A Gene Is Significantly Associated With Litter Size in Goat

**DOI:** 10.3389/fgene.2018.00091

**Published:** 2018-03-20

**Authors:** Yang Cui, Hailong Yan, Ke Wang, Han Xu, Xuelian Zhang, Haijing Zhu, Jinwang Liu, Lei Qu, Xianyong Lan, Chuanying Pan

**Affiliations:** ^1^College of Animal Science and Technology, Northwest A&F University, Yangling, China; ^2^Shaanxi Provincial Engineering and Technology Research Center of Cashmere Goats, Yulin University, Yulin, China; ^3^Life Science Research Center, Yulin University, Yulin, China

**Keywords:** cashmere goat, KDM6A gene, meiosis, mitosis, insertion/deletion (indel), litter size

## Abstract

A previous whole-genome association analysis identified *lysine demethylase 6A* (*KDM6A*), which encodes a type of histone demethylase, as a candidate gene associated to goat fecundity. *KDM6A* gene knockout mouse disrupts gametophyte development, suggesting that it has a critical role in reproduction. In this study, goat *KDM6A* mRNA expression profiles were determined, insertion/deletion (indel) variants in the gene identified, indel variants effect on *KDM6A* gene expression assessed, and their association with first-born litter size analyzed in 2326 healthy female Shaanbei white cashmere goats. *KDM6A* mRNA was expressed in all tissues tested (heart, liver, spleen, lung, kidney, muscle, brain, skin and testis); the expression levels in testes at different developmental stages [1-week-old (wk), 2, 3 wk, 1-month-old (mo), 1.5 and 2 mo] indicated a potential association with the mitosis-to-meiosis transition, implying that *KDM6A* may have an essential role in goat fertility. Meanwhile, two novel intronic indels of 16 bp and 5 bp were identified. Statistical analysis revealed that only the 16 bp indel was associated with first-born litter size (*P* < 0.01), and the average first-born litter size of individuals with an insertion/insertion genotype higher than that of those with the deletion/deletion genotype (*P* < 0.05). There was also a significant difference in genotype distributions of the 16 bp indel between mothers of single-lamb and multi-lamb litters in the studied goat population (*P* = 0.001). Consistently, the 16 bp indel also had a significant effect on *KDM6A* gene expression. Additionally, there was no significant linkage disequilibrium (LD) between these two indel loci, consistent with the association analysis results. Together, these findings suggest that the 16 bp indel in *KDM6A* may be useful for marker-assisted selection (MAS) of goats.

## Introduction

Improvements in female fertility are of critical importance for the goat industry. As one of the most important factors restricting female fertility, increasing litter size has received much more consideration (Naicy et al., 2016; Yang et al., 2017). However, litter size is a trait with low heritability in many livestock animals, including pigs (Córdoba et al., 2015) and goats (Shaat and Mäki-Tanila, 2009); therefore, traditional direct selection is ineffective. At present, marker-assisted selection (MAS), based on relevant genetic variants, is used extensively to improve traits with low heritability, such as those associated with growth and reproduction (Sharma et al., 2013; An et al., 2015; Tomas et al., 2016). To facilitate MAS application to litter size in the goat industry, critical genetic variants causing phenotypic advantage should be verified.

Currently, whole-genome sequencing and genome-wide association studies (GWAS) are used to explore genetic variants strongly associated with production traits (Lai et al., 2016; Mota et al., 2017; Wu et al., 2018); however, numerous potential genes identified by GWAS have not been fully verified. To address this problem, methods which combine GWAS analysis results and MAS to screen for critical genetic variations in large livestock populations have been developed. Previously, Hubert et al. (2014) used whole-genome re-sequencing to reveal that genomic variations within the bovine *transmembrane protein 95* (*TMEM95*) gene are associated with male reproductive performance. A genome scan in a French dairy goat population also found that variants in *diacylglycerol o-acyltransferase 1* (*DGAT1*) were associated with a notable decrease in milk fat content (Martin et al., 2017). These results demonstrate the feasibility of using combined methods to screen for important genetic variations.

In 2016, a study using whole-genome analysis to compare high and low fecundity groups of the Chinese Laoshan dairy goat identified several genes as potentially critical for fecundity, including *lysine demethylase 6A* (*KDM6A*), *androgen receptor* (*AR*), and *anti-Mullerian hormone receptor type 2* (*AMHR2*) (Lai et al., 2016). Among these genes, *KDM6A* encodes a protein that demethylates tri- and dimethylated lysine 27 of histone H3, and can affect gametophyte development. Importantly, numerous studies have verified that the *KDM6A* gene is vital for animal reproduction. In rodents, knock-out of the *KDM6A* gene disrupted primordial germ cell development (Mansour et al., 2012). In female mice, the Rhox cluster of genes, which contains reproduction-related homeobox genes, is also regulated by KDM6A (Berletch et al., 2013). Furthermore, *KDM6A* regulates maturation of the mouse oocyte (Xu et al., 2017). Overall, based on whole-genome analysis and rodent studies, there is strong evidence that *KDM6A* has crucial roles in modulation of goat fecundity.

To date, goat *KDM6A* gene expression profiles and DNA polymorphisms are largely unexplored. Therefore, in this study, the tissue expression profiles of the *KDM6A* gene were investigated, two novel indel variants in this gene identified and the relationship between these loci and first-born litter size evaluated in a large Shaanbei white cashmere goat population. Moreover, the relationship between the identified indel loci and *KDM6A* expression levels was assessed. Our findings provide a basis for further research about the underlying causal mutation and suggest hypotheses for further study leading to the application of MAS to goat breeding.

## Materials and methods

All experiments in this study involving animals were approved by the Faculty Animal Policy and Welfare Committee of Northwest A&F University (protocol number NWAFAC1008). Moreover, the care and use of experimental animals completely conformed with local animal welfare laws, guidelines, and policies.

### Sample collection

For DNA experiments, a total of 2,326 adult female Shaanbei white cashmere goats were randomly selected from a large population. These goats all received the same diet and were kept under standard conditions after weaning. Among these goats, 1,811 animals had records of first-born litter size data (Wang et al., 2017; Yang et al., 2017). Apart from these female goats, we also collected a total of 18 male goat samples from six different developmental periods for RNA experiments. Nine tissues (heart, liver, spleen, lung, kidney, testis, brain, skin, and muscle) were harvested from 1-week-old (wk) and 2-month-old (mo) male goats (*n* = 3 per group). Moreover, testes tissues samples were also collected at 2, 3 wk, 1, and 1.5 mo (*n* = 3 per group). All tissues were immediately frozen in liquid nitrogen and stored at −80°C.

### Isolation of DNA

Genomic DNA was isolated from ear tissues using the method published by Lan et al. (2007). The quality of genomic DNA samples was assayed using Nanodrop 2000 Spectrophotometer. DNA samples were each diluted to a working concentration of 10 ng/μL and stored at −20°C.

### Primer design, PCR amplification, and indel genotyping

Five primer pairs for amplification of indel loci in introns were designed using Primer Premier software (version 5.0) based on the goat *KDM6A* gene sequence (NW_017189516.1) and the NCBI SNP-database (https://www.ncbi.nlm.nih.gov/snp; Table 1). Assays were performed by touch-down PCR in a 13 μL volume, containing 6.5 μL 2 × mix, 0.3 μL each of forward and reverse primers, 0.8 μL genomic DNA (10 ng/μL), and 5.4 μL ddH_2_O. The PCR protocol was as follows: initial denaturation for 5 min at 95 °C; followed by 18 cycles of denaturation for 30 s at 94°C, annealing for 30 s at 68°C (with a decrease of 1°C per cycle), extension for 30 s at 72°C; another 25 cycles of 30 s at 94°C, 30 s at 50°C, and 2 min at 72°C; and a final extension for 10 min at 72°C, with subsequent cooling to 4°C. The genotyping of indel polymorphisms in goat *KDM6A* was performed by separation of PCR products (5 μL) by agarose gel electrophoresis.

**Table 1:** PCR primers used for detecting indel loci and qPCR analysis of goat *KDM6A* gene.

**Primer**	**Primer sequence (5′−3′)**	**Length**	**Function**
KDM6A-1F	CTGCACTTTGTCCAATGCTGA	132 bp	Indel detection
KDM6A-1R	AGATTCAGCAATTCCAGGGGA		
KDM6A-2F	GCAGCAGTAGAAATGGTC	162 bp	Indel detection
KDM6A-2R	CCCTATCTATTCTCACCC		
KDM6A-3F	AGAGTTCATTCACAGATTCCACTT	246 bp	Indel detection
KDM6A-3R	AAAAGAATCCAGGTGGGTGTCA		
KDM6A-4F	AATTTTGACACCCACCTGGA	186 bp	Indel detection
KDM6A-4R	CACTGAGCATGCAAAGGAATACA		
KDM6A-5F	TTGCTAGTTCCTTCTTCA	146 bp	Indel detection
KDM6A-5R	CCTCACTCAATTATTACATG		
KDM6Af	TGATCCCAGCTTTTGTCGAG	139 bp	qPCR
KDM6Ar	AGCATTGGACAAAGTGCAGG		
GAPDHf	AAAGTGGACATCGTCGCCAT	116 bp	Reference gene
GAPDHr	CCGTTCTCTGCCTTGACTGT		
ACTBf	CTGAGCGCAAGTACTCCGTGT	124 bp	Reference gene
ACTBr	GCATTTGCGGTGGACGAT		
RPL19f	GGGTACTGCCAATGCTCGAA	119 bp	Reference gene
RPL19r	TGTGATACATGTGGCGGTCA		

### Total RNA isolation and synthesis of cDNA

Total RNA was extracted from tissue samples using TRIzol total RNA extraction reagent (Takara, Dalian, China), according to the manufacturer's instructions. The integrity of total RNA was evaluated by 1% agarose gel electrophoresis in 6 × loading buffer (Takara, Dalian, China). The quantity and quality of total RNA was estimated using a Nanodrop 2000 Spectrophotometer with the OD_260_ nm/OD_280_ nm ratio expected to be between 1.8 and 2.0; meanwhile, the OD_260_ nm/OD_230_ nm ratio no less than 1.7 (Zhang et al., 2017). Samples were then stored at −80°C. Prime Script™ RT Reagent kit (Takara, Dalian, China) was used to synthesize first strand cDNA, according to the manufacturer's protocol. The resultant cDNA was stored at −20°C.

### Analysis of *KDM6A* mRNA expression profiles by quantitative real-time PCR

*KDM6A* gene expression profiles were analyzed by qPCR using cDNA from 1 wk and 2 mo male goat tissue samples. Expression profiles in testes at different time points (1, 2, 3 wk, 1, 1.5, and 2 mo) were also evaluated. qPCR primers were designed covering different exons in order to assure the amplification of the cDNA (Table 1). qPCR reactions (12 μL) contained 6 μL 2 × SYBR Premix ExTaq (Takara, Dalian, China), 0.5 μL of each primer, and 5 μL cDNA (1/100 dilution). PCR amplification was performed as follows: 95°C for 5 min followed by 40 cycles of 94°C for 30 s, 60°C for 30 s, and 72°C for 30 s (Yu et al., 2017). The expression levels of *RPL19* (ribosomal protein L19), *GAPDH* (glyceraldehyde-3-phosphate dehydrogenase) and *ACTB* (β-actin) in all isolated tissues were tested. The reference gene in each tissue was analyzed from the GeNorm program, which based on the *M*-values (reference gene with the lowest *M*-value is considered most stable; Vandesompele et al., 2002). After calculation, *RPL19* was used as the reference gene in lung, muscle, brain and skin. *ACTB* was used as the reference gene for evaluation of relative gene expression in heart, liver, spleen, kidney and testis. And previous studies also used *ACTB* to determine gene expression in goat testis (Yao et al., 2014; Deng et al., 2017b). The results were determined using the 2^−ΔΔCt^ method (Livak and Schmittgen, 2001).

### Statistical analysis

To explore the genetic structure of the indel variants in the investigated goat population, genetic diversity indices were calculated. The genotype and allele frequencies reflect the genetic composition of the indel variant in the tested goat population. Nei's methods were used to calculate population genetic diversity indices, including homozygosity (Ho), heterozygosity (He; Ho + He = 1) and polymorphism information content (PIC) (Nei and Roychoudhury, 1974). Ho and He are a measure of genetic variation of a population. PIC is an indicator of polymorphism. Based on PIC values, the genetic variations classified as high genetic diversity (PIC > 0.5), medium genetic diversity (0.25 < PIC < 0.5) and low genetic diversity (PIC < 0.25) (Botstein et al., 1980). The χ^2^ test using the SHEsis online platform (http://analysis.bio-x.cn) was conducted to evaluate HWE (Li et al., 2009; Chen et al., 2013).

Linkage disequilibrium (LD) is the nonrandom association of alleles at linked loci. In particular, many genetic variations correlated with each other due to LD; thus, LD plays a crucial role for mapping complex disease or trait-associated genes (Pritchard and Przeworski, 2001; Hazelett et al., 2016). Currently, to detect whether there is a linkage between the two indels identified in *KDM6A*, the LD structure as measured by *D'* and *r*^2^ were performed with the SHEsis online platform (http://analysis.bio-x.cn; Li et al., 2009). The *r*^2^-value was used as a pairwise measure of LD (Marty et al., 2010; Huang et al., 2015). The case of *r*^2^ = 0 is known as perfect LD, *r*^2^ > 0.33 indicates sufficiently strong LD, and *r*^2^ = 1 suggests complete LD (Ren et al., 2014).

Associations between indels and first-born litter size, to establish the influence of different parameters on litter size, were analyzed using a general linear model: Y_*ij*_ = μ + HYS_i_ + G_j_ + e_*ij*_, where *Y*_*ij*_ is the phenotypic value of litter size, μ is the overall population mean, HYS_*i*_ is the fixed effect of the herd-year-season, G_*j*_ is the fixed effect of genotype, and e_*ij*_ is the random error (Yang et al., 2017). The litter size data used in this study was first-born litter size; thus, the lambing year and parity were not included in the general linear model. The analysis was performed with SPSS 19.0 software by one-way ANOVA and compared using Tukey multiple test.

## Result

### mRNA expression profile of goat *KDM6A*

**Figure 1:**
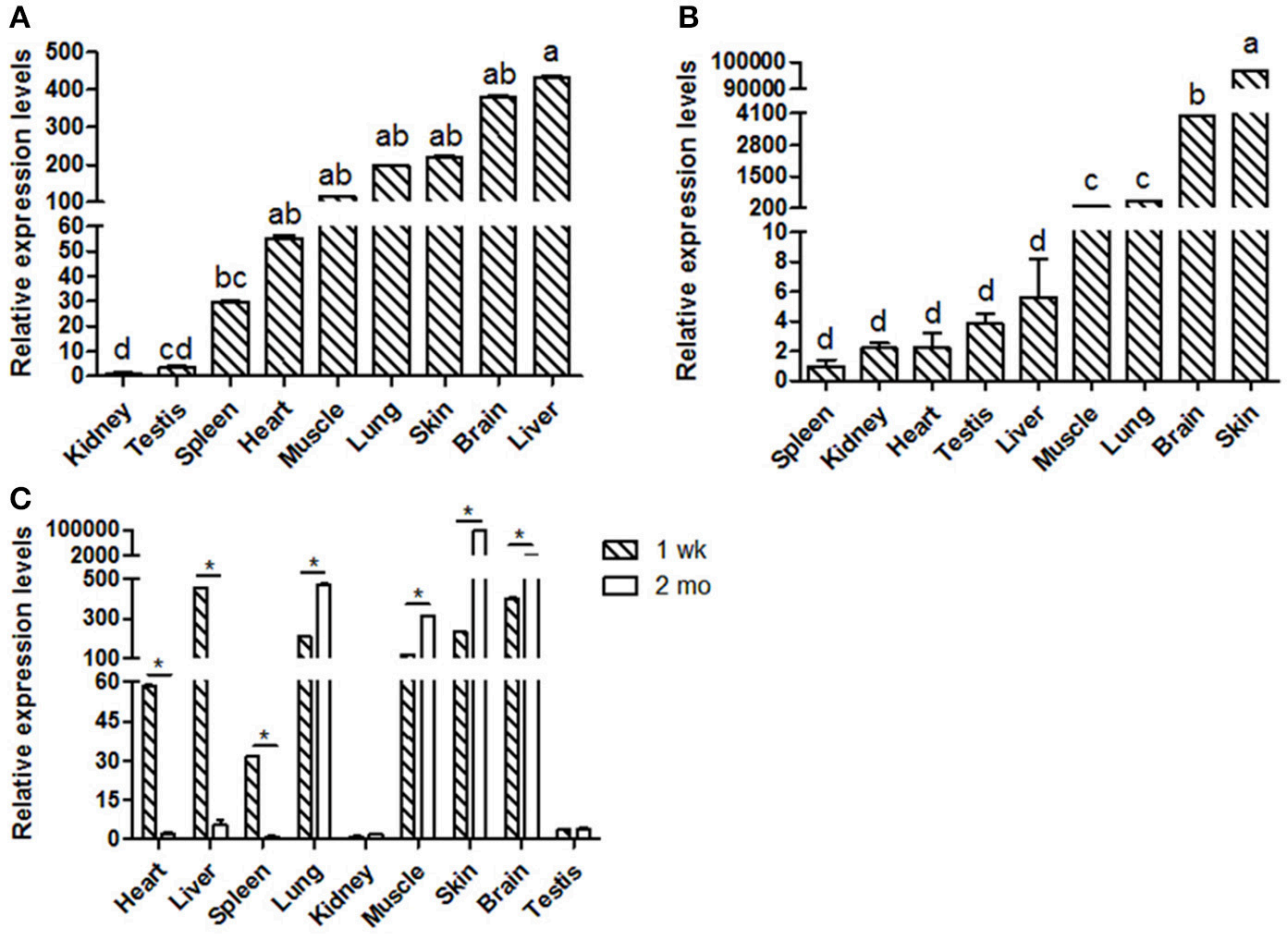
Shaanbei white cashmere goat *KDM6A* mRNA expression patterns detected by qPCR. **(A)** Different tissues of 1 wk Shaanbei white cashmere goat; **(B)** Different tissues of 2 mo Shaanbei white cashmere goat. **(C)** The comparison of goat *KDM6A* tissue expression levels between 1 wk and 2 mo. Data represent means ± SE (*n* = three samples of each tissues). Columns with different letters (a, b) means *P* < 0.05; ^*^P < 0.05.

**Figure 2:**
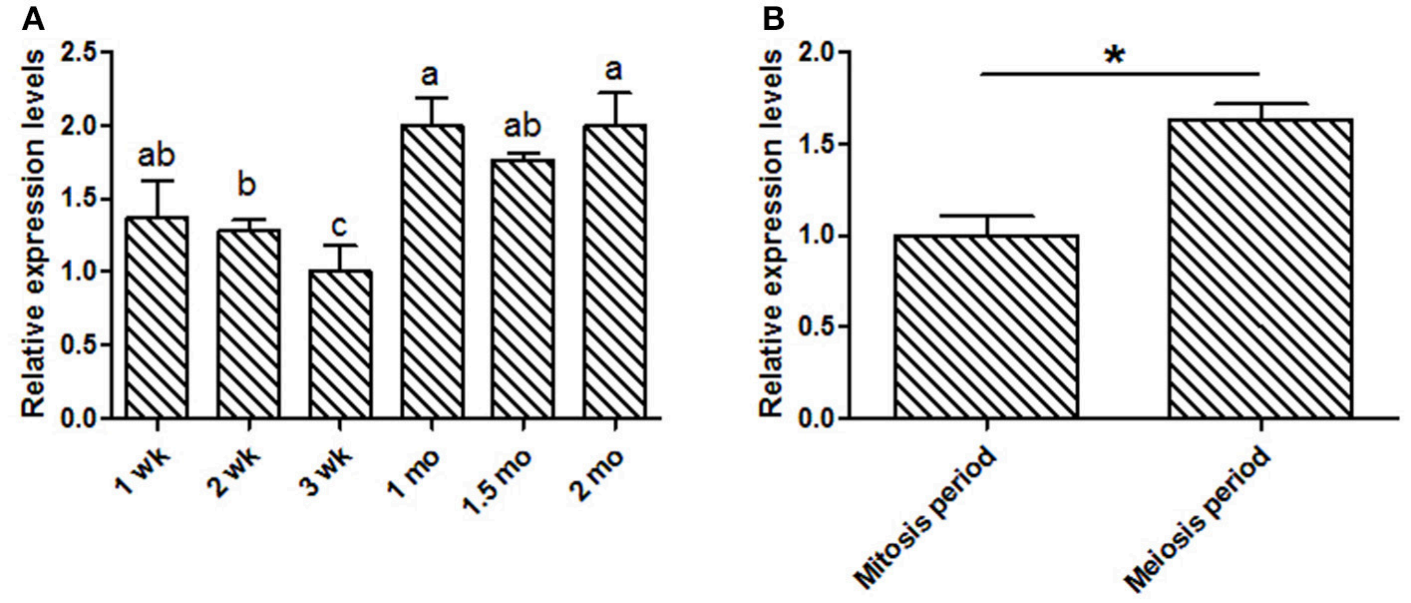
Expression of *KDM6A* mRNA was detected in goat testes tissues by qPCR. **(A)** *KDM6A* mRNA expression profiles at different stages in testis tissue. **(B)** Expression of *KDM6A* mRNA at mitosis and meiosis period in testis tissue. Data represent means ± SE (*n* = three samples per group). Columns with different letters (a, b) means *P* < 0.05; ^*^P < 0.05.

Goat *KDM6A* mRNA expression profiles were investigated in different tissues at 1 wk (Figure 1A) and 2 mo (Figure 1B). *KDM6A* was found to be expressed in all tissues tested at both developmental stages. Notably, the expression levels of *KDM6A* in heart, liver and spleen tissues were significantly higher at 1 wk than at 2 mo (*P* < 0.05). In contrast, the expression levels of *KDM6A* were significantly lower in lung, muscle, brain, and skin at 1 wk than at 2 mo (*P* < 0.05; Figure 1C).

### Goat *KDM6A* gene expression profiles in testis tissues

This study was focused on the reproductive system, thus the expression levels of *KDM6A* at different testis developmental stages (1, 2, 3 wk, 1, 1.5, and 2 mo) were explored. In testis tissues, the *KDM6A* mRNA expression levels at 2 and 3 wk were significantly lower than that at 1, 1.5, and 2 mo (*P* < 0.05; Figure 2A). In a previous study of Liaoning cashmere goat (the male parents of Shaanbei white cashmere goat) spermatogonia was found to be actively mitotic from the postnatal period, with primary spermatocytes, which result from meiosis, first appearing at 1 mo (Zhan, 2015). Thus, we divided testis development into two phases: birth to 1 mo, referred to as the mitosis period, and 1–2 mo, referred to as the meiosis period. *KDM6A* mRNA expression levels were significantly increased in meiosis period compared with the mitosis period (*P* < 0.05; Figure 2B). Together, these findings provide evidence that *KDM6A* has an important role in fertility. To explore potential DNA markers for improvement of goat fertility, we next focused on the identification of polymorphisms in *KDM6A*.

### Identification of genetic variants that regulate *KDM6A* expression

**Figure 3:**
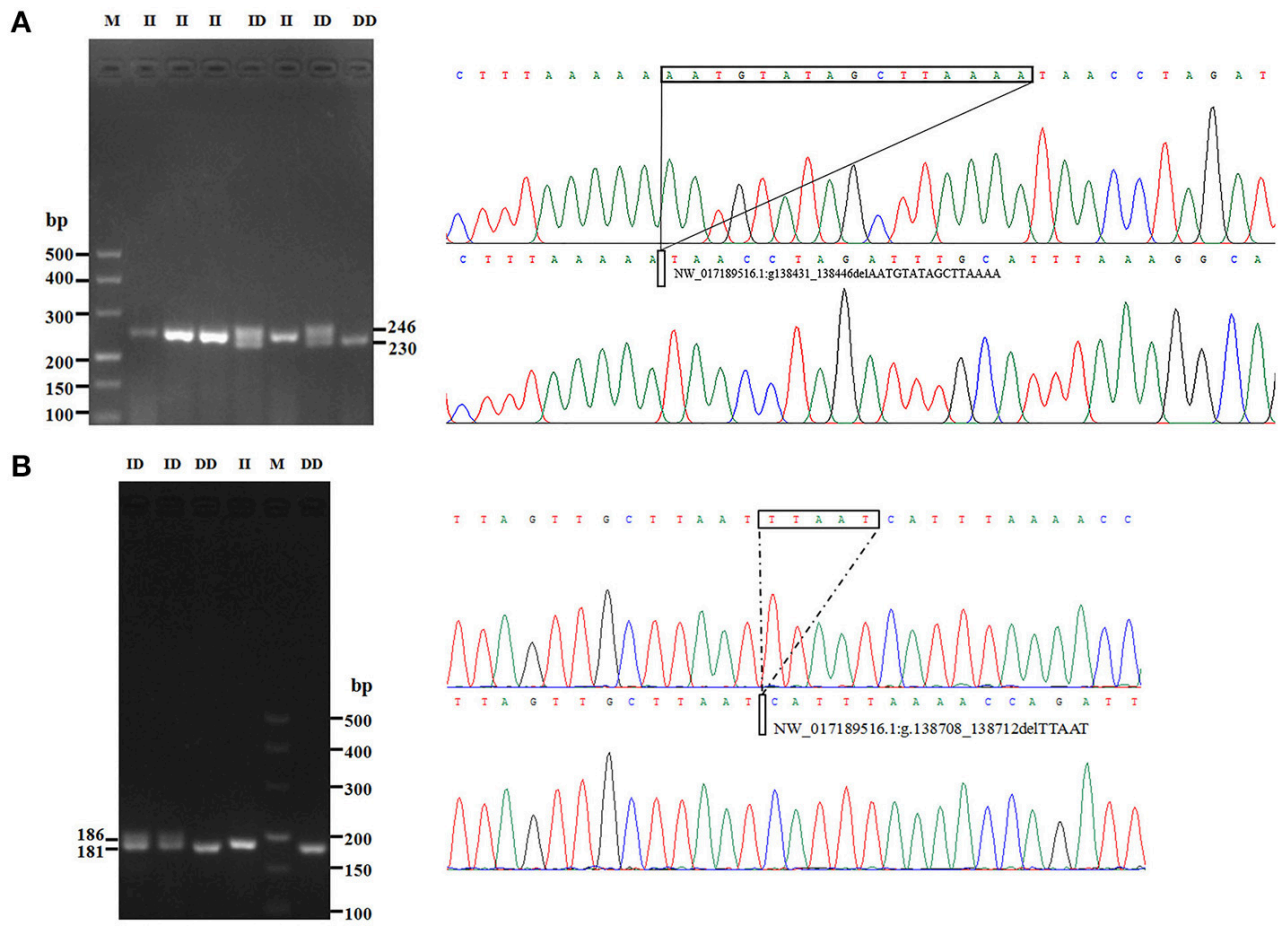
The electrophoresis diagrams and sequence diagrams of goat *KDM6A* gene indel loci. **(A)** 16 bp indel locus. **(B)** 5 bp indel locus.

In this study, two novel indel variants were detected in goat *KDM6A* introns; one of 16 bp indel (intron 17) (NW_017189516.1:g.138431_138446delAATGTATAGCTTAAAA; rs636691921) and another of 5 bp indel (intron 17) (NW_017189516.1:g.138708_138712delTTAAT; rs653321281). These indels were detected using primer 3 and 4, respectively. PCR products separated by agarose gel electrophoresis, and sequence diagrams of these two novel indels are presented in Figure 3 and Supplement Figure 1.

**Figure 4:**
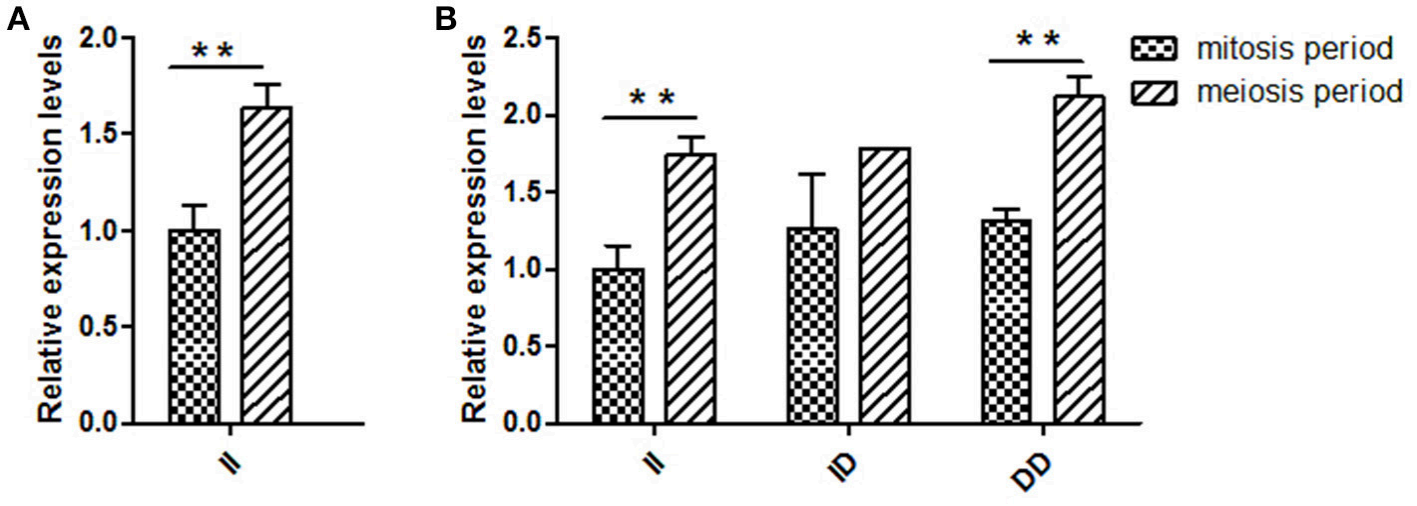
Two indel loci influence *KDM6A* mRNA expression between two periods. **(A)** In the 16 bp locus, *KDM6A* expression levels were significantly higher in the meiosis period of the II genotype. **(B)** In the 5 bp locus, the II and DD genotype had a significantly difference between two periods. Data represent means ± SE. ^**^P < 0.01.

Several previous studies have reported that variants in intron can affect gene transcription (Ren et al., 2011); therefore, *KDM6A* expression levels at different developmental periods were conducted in animals with the same genotype. However, in the mitosis period individuals that had DD and ID genotypes were not found at the 16 bp locus; thus, for this locus, we only compared the *KDM6A* expression levels of II genotype carriers between the mitosis and meiosis periods. The results demonstrated that *KDM6A* expression levels were significantly higher in the meiosis period of the II genotype at the 16 bp locus (*P* < 0.01). Furthermore, *KDM6A* expression was significantly higher during the meiosis period of animals with the II and DD genotypes of the 5 bp locus (*P* < 0.01; Figure 4). Together, these results indicate that the two indel loci could affect the expression levels of *KDM6A* and may influence the reproductive phenotype of goats. Therefore, the relationship between these indel loci and goat reproductive traits were further investigated in a large goat population.

### Genetic parameters and LD of the identified indel loci

**Figure 5:**
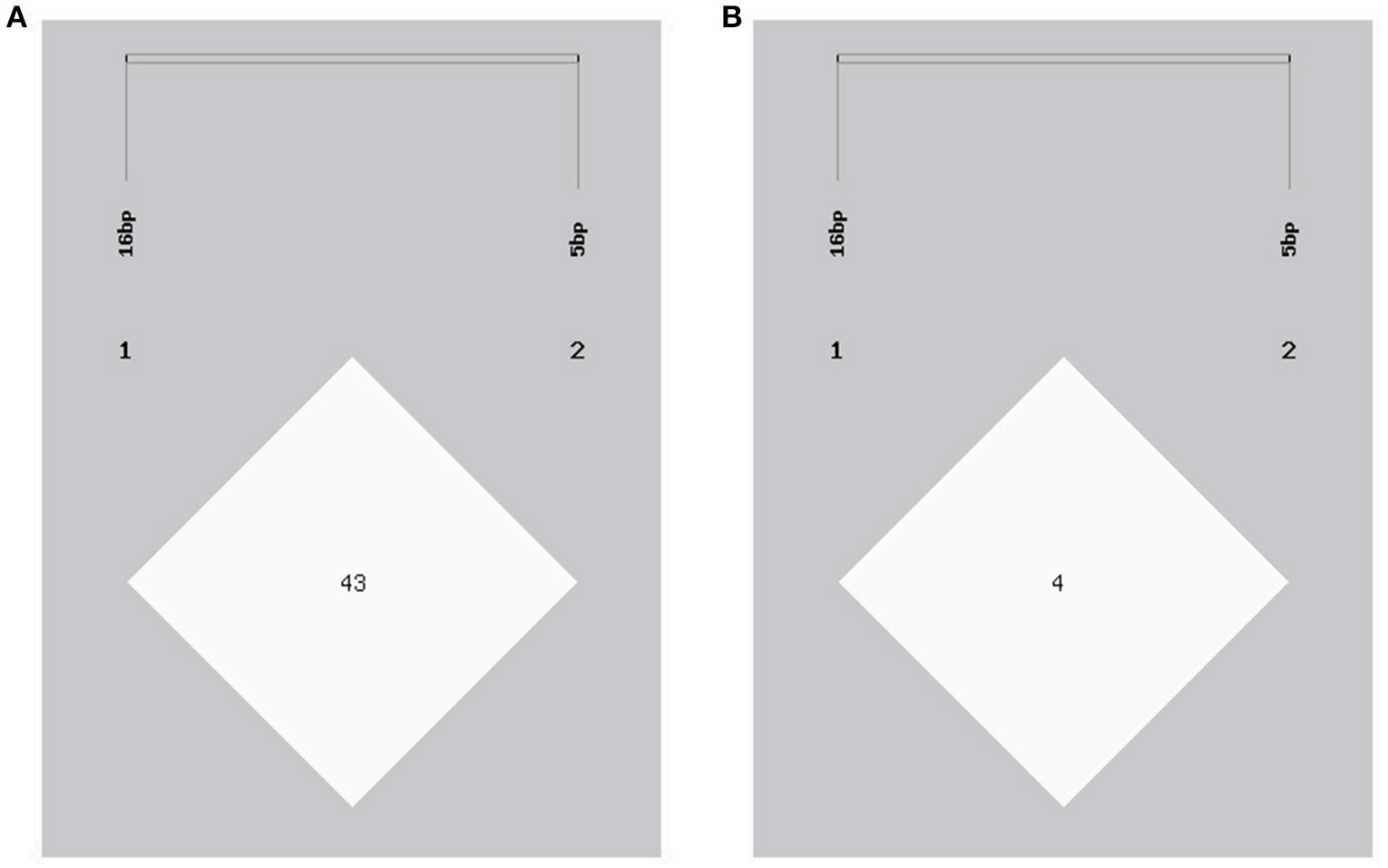
Linkage disequilibrium plot of the *KDM6A* gene two indel loci. **(A)** *D'* value, **(B)** *r*^2^ value.

The genotype and allele frequencies, as well as other genetic parameters, associated with the *KDM6A* indel loci were calculated to determine the genotype distribution among Shaanbei white cashmere goats (Table 2). The data indicated that “I” allele (0.941) of the 16 bp indel was more frequent than “D” allele (0.059). For the 5 bp indel, analysis of 615 individuals indicated that the frequency of the “I” allele was lower than 0.278, with the “D” allele present at a higher frequency (0.722). Additionally, the χ^2^ test indicated that the 5 bp indel genotype frequency was in agreement with HWE (*P* > 0.05) in the Shaanbei cashmere goat population; however, the 16 bp indel did not conform to HWE (*P* < 0.05; Table 2). Based on PIC values, the 16 bp locus had low genetic diversity (PIC = 0.105), and the 5 bp locus had medium genetic diversity (PIC = 0.321). Moreover, we analyzed the LD between these two indel loci; however, no LD was detected between them (*r*^2^ = 0.047; Table 3; Figure 5).

**Table 2:** Genetic parameters of the 16 and 5 bp loci within *KDM6A* gene in Shaanbei white cashmere goat.

**Loci**	**Observed genotypes (numbers)**	**Frequencies**		**Ho**	**He**	**PIC**	**χ^2^ (*P*-value)**
		**Genotypes**	**Alleles**				
16-bp (*n* = 2,326)	II (2074)	0.892	0.941 (I)	0.889	0.111	0.105	27.155 (*P* = 1.269E-06)
	ID (230)	0.099	0.059 (D)				
	DD (22)	0.009					
5-bp (*n* = 615)	II (52)	0.085	0.278 (I)	0.598	0.401	0.321	0.800 (*P* = 0.670)
	ID (238)	0.387	0.722 (D)				
	DD (325)	0.528					

**Table 3:** Estimated values of linkage equilibrium analysis for two indels in *KDM6A* gene in studied populations.

**Indel**	***D'***	***r^2^***
5 bp/16 bp	0.432	0.047

### Analyses of associations between indel variations and first-born litter size

Next, the associations between *KDM6A* indel loci and there productive performance of female goats (first-born litter size) were investigated. The results showed that there was no relationship between the 5 bp indel and first-born litter size in populations of different sizes (*n* = 300–600 individuals) randomly selected from the whole population (*P* > 0.05; Table 4). Notably, the 16 bp locus was always associated with first-born litter size (*P* < 0.01) from 300 to 600 and even reaching 1811 individuals, with animals with the II genotype having larger first-born litter size than those with the DD genotype (Table 4).

**Table 4:** Associations of the 16 and 5 bp loci with first-born litter size in detected groups with different numbers.

**Number**	**Genotypes (16 bp)**			***P*-values**	**Genotypes (5 bp)**			***P*-values**
	**II**	**ID**	**DD**		**II**	**ID**	**DD**	
100	1.49 ± 0.05^b^	1.90 ± 0.10^a^	1.00 ± 0.00^c^	**0.001**	1.71 ± 0.18^a^	1.69 ± 0.07^a^	1.33 ± 0.06^b^	**0.001**
200	1.52 ± 0.04^a^	1.54 ± 0.10^a^	1.00 ± 0.00^b^	**0.015**	1.58 ± 0.15^b^	1.63 ± 0.06^a^	1.41 ± 0.05^b^	**0.010**
300	1.53 ± 0.04^a^	1.51 ± 0.08^a^	1.00 ± 0.00^b^	**0.010**	1.55 ± 0.11	1.56 ± 0.05	1.45 ± 0.04	0.179
400	1.54 ± 0.03^a^	1.50 ± 0.07^a^	1.00 ± 0.00^b^	**0.002**	1.54 ± 0.09	1.52 ± 0.04	1.48 ± 0.04	0.647
500	1.54 ± 0.03^a^	1.53 ± 0.06^a^	1.00 ± 0.00^b^	**0.001**	1.51 ± 0.08	1.52 ± 0.04	1.48 ± 0.03	0.818
607	1.55 ± 0.03^a^	1.45 ± 0.06^a^	1.00 ± 0.00^b^	**2.54E-04**	1.49 ± 0.07	1.46 ± 0.03	1.45 ± 0.03	0.812
800	1.54 ± 0.02^a^	1.45 ± 0.05^a^	1.00 ± 0.00^b^	**8.83E-07**				
1000	1.54 ± 0.02^a^	1.44 ± 0.04^a^	1.00 ± 0.00^b^	**9.93E-07**				
1200	1.46 ± 0.02^a^	1.41 ± 0.04^a^	1.06 ± 0.06^b^	**0.003**				
1811	1.49 ± 0.01^a^	1.43 ± 0.04^a^	1.06 ± 0.06^b^	**0.001**				

*Data represent means ± SE. Cells with different letters (a, b) means P < 0.05. Bold values mean P < 0.05*.

Furthermore, we investigated the genotype distributions of these two indel loci in groups of goats with first-born single-lamb and multi-lamb litters, using the same test groups described above (*n* = 100–600 individuals; Tables 5, 6). The results demonstrate that only the 16 bp indel had different genotype distributions between the two groups of goats with different litter types (*P* < 0.01). These results were consistent with those of association analyses; therefore, we tested the 16 bp indel in a total of 1,811 individuals. The results indicated a significant difference in genotype distributions between groups with first-born single-lamb and multi-lamb litters at the 16 bp indel (*P* = 0.001; Table 5). There was no LD between the two indel loci, consistent with the results of the association analysis (Figure 5).

**Table 5:** The 16 bp locus genotype distribution between mothers of single lamb and multi-lamb litters in Shaanbei white cashmere goats.

**Number**	**MSL genotypes (frequencies)**			**MML genotypes (frequencies)**			***P*-value**
	**II**	**ID**	**DD**	**II**	**ID**	**DD**	
100	42(0.84)	1(0.02)	7(0.14)	41(0.82)	9(0.18)	0	**0.001**
200	81(0.81)	11(0.11)	8(0.08)	87(0.87)	13(0.13)	0	**0.015**
300	119(0.793)	22(0.147)	9(0.06)	129(0.86)	21(0.14)	0	**0.009**
400	156(0.78)	33(0.165)	11(0.055)	171(0.855)	29(0.145)	0	**0.003**
500	200(0.833)	27(0.113)	13(0.054)	213(0.852)	37(0.148)	0	**0.001**
600	236(0.787)	50(0.166)	14(0.047)	262(0.873)	38(0.127)	0	**2.04E-04**
800	317(0.793)	69(0.173)	14(0.034)	347(0.868)	53(0.132)	0	**1.62E-04**
1000	402(0.804)	83(0.166)	15(0.03)	438(0.876)	62(0.124)	0	**5.59E-05**
1200	559(0.832)	97(0.144)	16(0.024)	463(0.877)	64(0.121)	1(0.002)	**0.003**
1811	836(0.870)	108(0.112)	17(0.018)	771(0.907)	78(0.092)	1(0.001)	**0.001**

*MSL, Mothers of single lamb, MML, Mothers of multi-lamb (≥2). Bold values mean P < 0.05*.

**Table 6:** The 5 bp locus genotype distribution between mothers of single lamb and multi-lamb litters in Shaanbei white cashmere goats.

**Number**	**MSL genotypes (frequencies)**			**MML genotypes (frequencies)**			***P*-value**
	**II**	**ID**	**DD**	**II**	**ID**	**DD**	
100	2(0.040)	12(0.240)	36(0.720)	5(0.143)	12(0.343)	18(0.514)	**0.001**
200	5(0.050)	27(0.270)	68(0.680)	7(0.070)	46(0.460)	47(0.470)	**0.010**
300	9(0.060)	51(0.340)	90(0.600)	11(0.073)	65(0.433)	74(0.493)	0.178
400	15(0.075)	74(0.370)	111(0.555)	19(0.095)	78(0.39)	103(0.515)	0.646
500	22(0.088)	94(0.376)	134(0.536)	24(0.096)	99(0.396)	127(0.508)	0.817
600	26(0.079)	129(0.391)	175(0.530)	26(0.093)	106(0.380)	147(0.527)	0.811

*MSL, Mothers of single lamb; MML, Mothers of multi-lamb (≥2). Bold values mean P < 0.05*.

### Influence of the 16 Bp indel on *KDM6A* expression during the meiosis period

**Figure 6:**
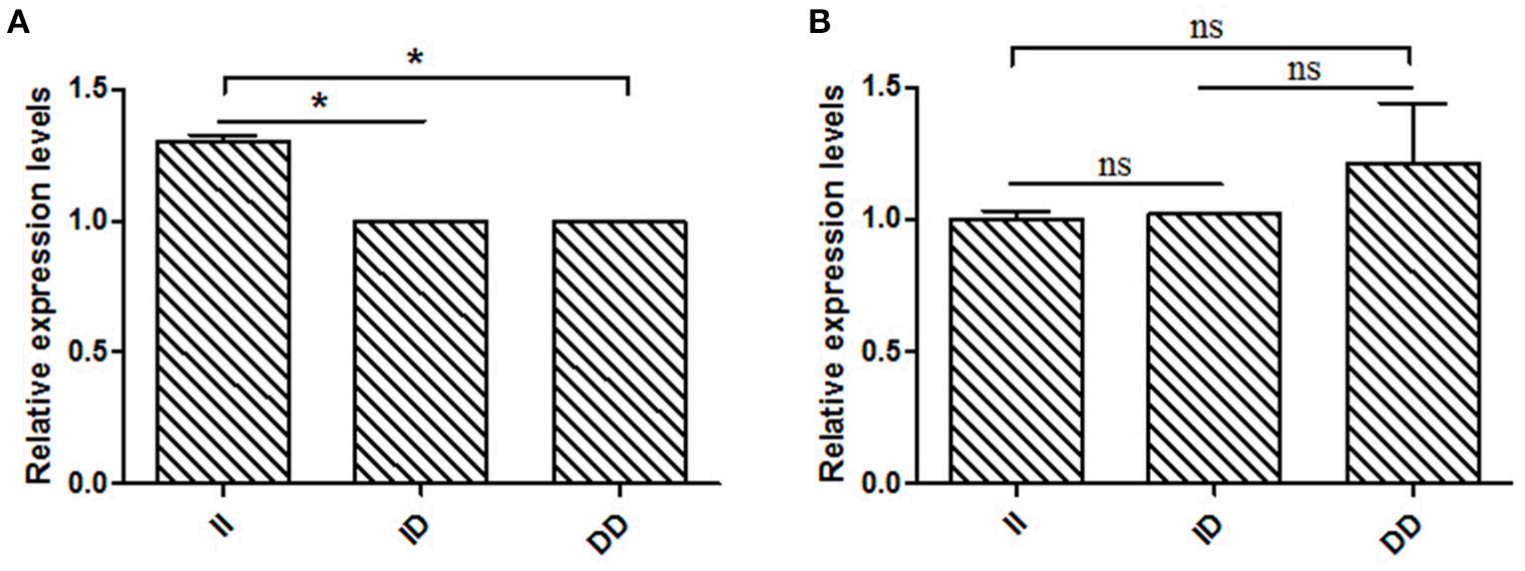
The 16 bp indel locus influence *KDM6A* expression in meiosis period. **(A)** In the 16 bp locus, genotype II had a significantly higher *KDM6A* expression level than genotype ID and DD. **(B)** In the 5 bp locus, there was non-difference among different genotypes. Data represent means ± SE. ^*^P < 0.05; ns, no significance.

Based on the results of the association analyses, we hypothesized that the 16 bp indel can influence goat reproductive phenotype. This phenomenon may be attributable to the effect of genotype at this locus on *KDM6A* mRNA expression levels. Therefore, we tested *KDM6A* mRNA expression levels in testis tissue from animals with three genotypes at the two indel loci during the meiosis period. At the 16 bp indel locus, the individuals with II genotype had significantly higher levels of *KDM6A* mRNA expression than those with the ID and DD genotype (*P* < 0.05; Figure 6); however, there were no significant differences in *KDM6A* expression levels among different genotypes at the 5 bp indel locus (*P* > 0.05; Figure 6).

## Discussion

Previously, Lai et al. (2016) determined that variants of the *KDM6A* gene were closely related to fecundity in Laoshan dairy goats using deep sequencing analysis. Several studies have also explored the role of *KDM6A* in reproductive biology (Yap et al., 2011), and their findings suggest that this gene has an essential role in reproduction. However, there are no previous reports of goat *KDM6A* tissue expression profiles. The relationship between *KDM6A* gene variants and first-born litter size in large Shaanbei white cashmere goat population (*n* = 2,326) required further investigation.

First, we determined the expression profiles of the goat *KDM6A* gene, and the results demonstrated that it was widely expressed in various organs. As the *KDM6A* gene is reported to be associated with spermatogenesis (Teperek et al., 2016), we next determined its expression patterns at different developmental stages in testis. Interestingly, the mRNA expression levels of *KDM6A* at later developmental stages (1, 1.5, and 2 mo) were higher than those at earlier stages (1, 2, and 3 wk). A study of the Liaoning cashmere goat reported that their spermatogonia gradually proliferate via mitotic division from birth, and primary spermatocyte development, which initiate meiosis from 1 mo (Zhan, 2015). Therefore, we combined individuals at 1, 2, and 3 wk classified as the mitosis period; similarly the 1, 1.5, and 2 mo data were considered the meiosis period. Our results demonstrate that *KDM6A* mRNA expression levels during the mitosis stage were lower than those in the meiosis stage (*P* < 0.05), suggesting *KDM6A* may be associated with the mitosis-to-meiosis transition in the Shaanbei white cashmere goat. Additionally, previous reports indicate that KDM6A regulates oocyte meiosis resumption in female mice, and abnormal expression of this gene causes aberrant H3K27me3, leading to disruption of oocyte maturation (Xu et al., 2017). Together, these data indicated that the *KDM6A* gene may have an essential role in meiosis resumption in both male and female animals.

In addition to the *KDM6A* gene, deep sequencing analyses of the Laoshan dairy goat have also identified genetic variants in male sex differentiation genes, including *AR* and *AMHR2* that are closely associated with female fecundity (Lai et al., 2016). Furthermore, with the development of modern and intensive breeding condition, the number of male livestock is far less than the female (Wang et al., 2017). We hoped to explore the genetic variation in goat *KDM6A*, with the aim of implementing the identified polymorphisms as molecular markers to contribute to MAS in goat breeding. Therefore, we performed further analysis of *KDM6A* genetic effects in the female Shaanbei white cashmere goat population.

Currently, natural genetic variations are divided into three forms: SNPs (single nucleotide polymorphisms), indels and SVs (larger structural variants; Julienne et al., 2010). Unlike other genetic variations, indels can be directly detected by simple PCR amplification and agarose gel electrophoresis, making them convenient and practical (Naicy et al., 2016). Therefore, indel variants in the *KDM6A* gene were identified and their associations with first-born litter size investigated in a large commercial population of 2,326 Shaanbei white cashmere goats. Two novel indel loci (16 and 5 bp) were identified in putative intron 17 sequences, and each had three genotypes (II, ID, and DD). The 5 bp indel was in HWE (*P* > 0.05); however, the 16 bp locus was not (*P* < 0.05), because of the lower number of observed DD genotypes. One possible reason for this is rapid, powerful, and effective selection, which could affect the allelic balance of the indel locus (Zhao et al., 2013; Wang et al., 2015). Therefore, our data indicate that the selection pressure was more powerful on 16 bp than the 5 bp indel locus in the investigated goat population.

To analyze the association between indel loci and first-born litter size, we developed a novel strategy. Initially, analysis of the two indel loci was investigated in the same groups of 100–600 individuals, which were selected randomly from the whole population. When an indel locus in any investigated subset shows significant correlation with phenotype, it can be considered that this site is indeed correlated with the tested trait, especially in large population. This strategy improves the credibility of the test. Using groups of 300–600 individuals, there was no relationship between first-born litter size and the 5 bp indel locus (*P* > 0.05; Table 4). Interestingly, the 16 bp locus was consistently associated with first-born litter size in the same test groups (*P* < 0.01). Based on this data, we performed further analysis of the 16 bp indel among all individuals, and found that the association with first-born litter size was retained (*P* < 0.01), with the II genotype associated with larger litter size relative to the other genotypes, suggesting that the allele “I” of the *KDM6A* gene positively effects fecundity in this breed. Next, we adopted the same strategy to compare genotype distributions at these two indel loci between females who had first-born single-lamb and multi-lamb litters. The analysis results indicate that the 16 bp indel was very strongly associated with goat first-born litter size. Compared with direct analysis in the tested population (Deng et al., 2017a), this new strategy may provide more detailed and reliable results of association analysis. Additionally, the 16 bp locus had a significant effect on *KDM6A* gene expression, further implying a huge potential application for analysis of this locus. Moreover, linkage analysis demonstrated no LD between the two analyzed indel loci, consistent with the different results of association analyses.

**Figure 7:**
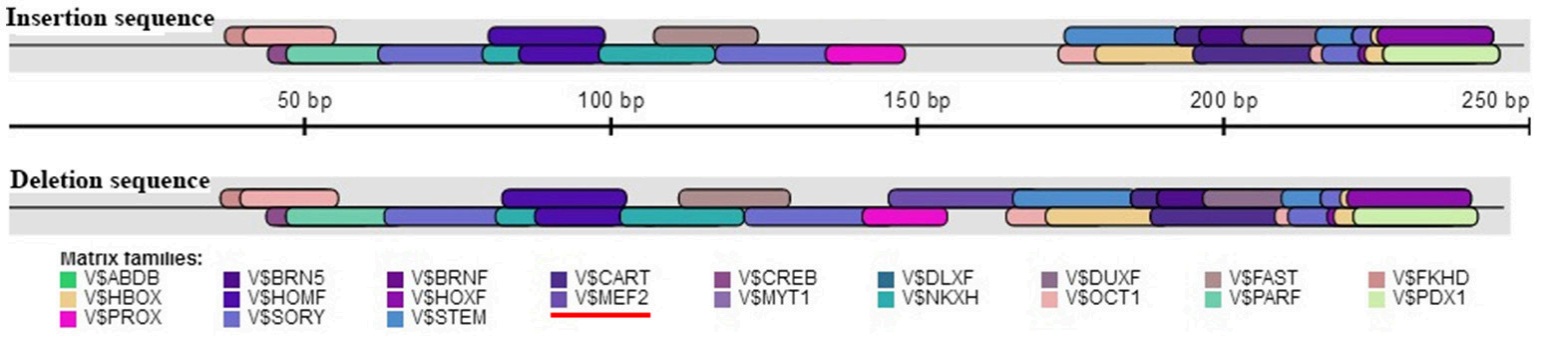
Bioinformatics predict transcription factor binding sites on the 16 bp indel sequences. One potential transcriptional factor MEF2 (myocyte-specific enhancer factor 2) only appear at deletion sequence. Red underline highlights MEF2 factor.

The association analysis based on the large experimental population revealed that the 16 bp indel located in the 17 intron of *KDM6A* was strongly associated with litter size in goats, which was consistent with the previous whole-genome analysis for Laoshan dairy goats (Lai et al., 2016). Since Ren et al. (2011) reported that intronic variations could affect the gene expression level, the relationship between the 16 bp indel and the expression of *KDM6A* was evaluated in the current study. Our results showed that the intronic 16 bp indel significantly associated with the expression of *KDM6A*. According to previous investigations, the intronic variations could impact the interaction between transcription factors and host genes (Van Laere et al., 2003; Fushan et al., 2009; Soldner et al., 2016). Therefore, the transcription factor binding site on the 16 bp indel sequence was predicted using the online software Genomatix MatInspector (http://www.genomatix.de; Cartharius et al., 2005). The bioinformatics analysis results showed that myocyte-specific enhancer factor 2 (MEF2), as transcription factor, could bind to the sequence in the context of lacking the 16 bp nucleotides (Figure 7). This discovery provided a possibility that MEF2 factor influence goat litter size. However, in mouse, MEF2 was expressed in the testis throughout development but absent in the ovary (Daems et al., 2014), which meant the impact of the 16 bp indel on litter size might not caused by MEF2 factor. In addition, some intronic variations could be in perfect LD with known phenotype-associated mutations (Nakaoka et al., 2016). For example, a 40 bp indel variant residing in the *mouse double minute 2 homolog* (*MDM2*) gene promoter is in complete LD with a SNP (rs2279744), and the SNP locus has been demonstrated to be associated with the susceptibility to several cancers. Through linkage with the SNP locus, this indel locus had positive association with risk of colon cancer (Gansmo et al., 2016). Of course, whether the 16 bp intronic indel influences phenotype through linkage with causal mutations need further study to be proven.

## Conclusion

In this study, the results indicated that goat *KDM6A* mRNA was expressed in all tissues tested (heart, liver, spleen, lung, kidney, muscle, brain, skin, and testis), and the expression levels in testis were significantly increased through mitosis-to-meiosis transition. Meanwhile, two novel intronic indels of 16 and 5 bp were identified, and only the 16 bp indel was significantly associated with first-born litter size (*P* < 0.01). Additionally, the 16 bp indel had a significant effect on *KDM6A* gene expression. These findings would provide a basis for further research about the underlying causal mutation and the application of MAS to goat breeding.

## Ethics statement

On the basis of experimental animal management measures in Shaanxi province (016000291szfbgt-2011-000001), all experiment procedures were approved by the Review Committee for the Use of Animal Subjects of Northwest A&F University. Animal experimentation, including sample collection, was performed in agreement with the ethical commission's guidelines.

## Author contributions

YC, XL, and CP came with idea and wrote manuscript. KW, HX, JL, HZ, and LQ collected the goat samples and isolated of genomic DNA. YC, HY, and XZ performed the experiments. YC, HY, and HX analyzed the data. All authors approved the final version of the manuscript for submission.

### Conflict of interest statement

The authors declare that the research was conducted in the absence of any commercial or financial relationships that could be construed as a potential conflict of interest.

## Supplementary material

Supplement Figure 1 The original image of the electrophoresis diagrams. **(A)** 16 bp indel locus. **(B)** 5 bp indel locus.

## Abbreviations

KDM6A: lysine-specific demethylase 6A
indel: insertion/deletion
SNPs: single nucleotide polymorphisms
LD: linkage disequilibrium
MAS: marker-assisted selection
Ho: homozygosity
He: heterezygosity
PIC: polymorphism information content
HWE: Hardy-Weinberg equilibrium
II: insertion/insertion
ID: insertion/deletion
DD: deletion/deletion
wk: week-old
mo: month-old
LD: linkage disequilibrium
MSL: Mothers of single lamb
MML: Mothers of multi-lamb.

## References

[B1] An X. Hou J. Gao T. Lei Y. Li G. Song Y.. (2015). Single-nucleotide polymorphisms g.151435C> T and g.173057T> C in PRLR gene regulated by bta-miR-302a are associated with litter size in goats. Teriogenology 83, 1477–1483. 10.1016/j.theriogenology.2015.01.030 25799469

[B2] Berletch J. B. Deng X. Nguyen D. K. Disteche C. M. (2013). Female bias in Rhox6 and 9 regulation by the histone demethylase KDM6A. PLoS Genet. 9:e1003489. 10.1371/journal.pgen.1003489 23658530 PMC3642083

[B3] Botstein D. White R. L. Skolnick M. Davis R. W. (1980). Construction of a genetic linkage map in man using restriction fragment length polymorphisms. Am. J. Hum. Genet. 32, 314–331. 6247908 PMC1686077

[B4] Cartharius K. Frech K. Grote K. Klocke B. Haltmeier M. Klingenhoff A.. (2005). MatInspector and beyond: promoter analysis based on transcription factor binding sites. Bioinformatics 21, 2933–2942. 10.1093/bioinformatics/bti473 15860560

[B5] Chen F. Shi J. Luo Y. Q. Sun S. Y. Pu M. (2013). Genetic characterization of the gypsy moth from China (*Lepidoptera, Lymantriidae*) using inter simple sequence repeats markers. PLoS ONE 8:e73017. 10.1371/journal.pone.0073017 23951339 PMC3737146

[B6] Córdoba S. Balcells I. Castelló A. Ovilo C. Noguera J. L. Timoneda O.. (2015). Endometrial gene expression profile of pregnant sows with extreme phenotypes for reproductive efficiency. Sci. Rep. 5:14416. 10.1038/srep14416 26435523 PMC5155628

[B7] Daems C. Martin L. J. Brousseau C. Tremblay J. J. (2014). MEF2 is restricted to the male gonad and regulates expression of the orphan nuclear receptor NR4A1. Mol. Endocrinol. 28, 886–898. 10.1210/me.2013-1407 24694307 PMC5414842

[B8] Deng S. L. Zhang Y. Yu K. Wang X. X. Chen S. R. Han D. P.. (2017a). Melatonin up-regulates the expression of the GATA-4 transcription factor and increases testosterone secretion from Leydig cells through RORα signaling in an *in vitro* goat spermatogonial stem cell differentiation culture system. Oncotarget 8, 110592–110605. 10.18632/oncotarget.22855 29299171 PMC5746406

[B9] Deng T. Pang C. Ma X. Duan A. Liang S. Lu X.. (2017b). Buffalo SREBP1: molecular cloning, expression and association analysis with milk production traits. Anim. Genet. 48, 720–721. 10.1111/age.12587 29023847

[B10] Fushan A. A. Simons C. T. Slack J. P. Manichaikul A. Drayna D. (2009). Allelic polymorphism within the TAS1R3 promoter is associated with human taste sensitivity to sucrose. Curr. Biol. 19, 1288–1293. 10.1016/j.cub.2009.06.015 19559618 PMC2742917

[B11] Gansmo L. B. Vatten L. Romundstad P. Hveem K. Ryan B. M. Harris C. C.. (2016). Associations between the MDM2 promoter P1 polymorphism del1518 (rs3730485) and incidence of cancer of the breast, lung, colon and prostate. Oncotarget 7, 28637–28646. 10.18632/oncotarget.8705 27081698 PMC5053751

[B12] Hazelett D. J. Conti D. V. Han Y. Al Olama A. A. Easton D. Eeles R. A.. (2016). Reducing GWAS complexity. Cell Cycle 15, 22–24. 10.1080/15384101.2015.1120928 26771711 PMC4825730

[B13] Huang Y. Z. Jing Y. J. Sun Y. J. Lan X. Y. Zhang C. L. Song E. L.. (2015). Exploring genotype-phenotype relationships of the LHX3 gene on growth traits in beef cattle. Gene 561, 219–224. 10.1016/j.gene.2015.02.030 25688878

[B14] Hubert P. Sabine K. Christine W. Hermann S. Reiner E. Sandra J. (2014). A nonsense mutation in TMEM95 encoding a nondescript transmembrane protein causes idiopathic male subfertility in cattle. PLoS Genet. 10:e1004044 10.1371/journal.pgen.1004044 24391514 PMC3879157

[B15] Julienne M. M. Ryan E. M. Scott E. D. (2010). Small insertions and deletions (INDELs) in human genomes. Hum. Mol. Genet. 19, R131–R136. 10.1093/hmg/ddq400 20858594 PMC2953750

[B16] Lai F. N. Zhai H. L. Cheng M. Ma J. Y. Cheng S. F. Ge W.. (2016). Whole-genome scanning for the litter sizetrait associated genes and SNPs under selection in dairy goat (*Capra hircus*). Sci. Rep. 6:38096. 10.1038/srep38096 27905513 PMC5131482

[B17] Lan X. Y. Pan C. Y. Chen H. Zhang C. L. Li J. Y. Zhao M. (2007). An AluI PCR-RFLP detecting a silent allele at the goat POU1F1 locus and its association with production traits. Small. Ruminant. Res. 73, 8–12. 10.1016/j.smallrumres.2006.10.009

[B18] Li Z. Zhang Z. He Z. Tang W. Li T. Zeng Z.. (2009). A partition–ligation–combination–subdivision EM algorithm for haplotype inference with multiallelic markers: update of the SHEsis. Cell Res. 19, 519–523. 10.1038/cr.2009.33 19290020

[B19] Livak K. J. Schmittgen T. D. (2001). Analysis of relative gene expression data using real-time quantitative PCR and the 2(-Delta Delta C (T)) Method. Methods 25, 402–408. 10.1006/meth.2001.1262 11846609

[B20] Mansour A. A. Gafni O. Weinberger L. Zviran A. Ayyash M. Rais Y.. (2012). The H3K27 demethylaseUtx regulates somatic and germ cell epigenetic reprogramming. Nature 488, 409–413. 10.1038/nature11272 22801502

[B21] Martin P. Palhière I. Maroteau C. Bardou P. Canale-Tabet K. Sarry J.. (2017). A genome scan for milk production traits in dairy goats reveals two new mutations in Dgat1 reducing milk fat content. Sci. Rep. 7:1872. 10.1038/s41598-017-02052-0 28500343 PMC5431851

[B22] Marty A. Amigues Y. Servin B. Renand G. Levéziel H. Rocha D. (2010). Genetic variability and linkage disequilibrium patterns in the bovine DNAJA1 gene. Mol. Biotechnol. 44, 190–197. 10.1007/s12033-009-9228-y 20012712

[B23] Mota R. R. Guimarães S. E. Fortes M. R. Hayes B. Silva F. F. Verardo L. L.. (2017). Genome-wide association study and annotating candidate gene networks affecting age at first calving in Nellore cattle. J. Anim. Breed. Genet. 134, 484–492. 10.1111/jbg.12299 28994157

[B24] Naicy T. Venkatachalapathy R. T. Aravindakshan T. V. Radhika G. Raghavan K. C. Mini M.. (2016). Nerve Growth Factor gene ovarian expression, polymorphism identification, and association with litter size in goats. Theriogenology 86, 2172–2178. 10.1016/j.theriogenology.2016.07.011 27544869

[B25] Nakaoka H. Gurumurthy A. Hayano T. Ahmadloo S. Omer W. H. Yoshihara K.. (2016). Allelic imbalance in regulation of ANRIL through chromatin interaction at 9p21 endometriosis risk locus. PLoS Genet. 12:e1005893. 10.1371/journal.pgen.1005893 27055116 PMC4824487

[B26] Nei M. Roychoudhury A. K. (1974). Sampling variances of heterozygosity and genetic distance. Genetics 76, 379–390. 4822472 10.1093/genetics/76.2.379 PMC1213072

[B27] Pritchard J. K. Przeworski M. (2001). Linkage disequilibrium in humans: models and data. Am. J. Hum. Genet. 69, 1–14. 10.1086/321275 11410837 PMC1226024

[B28] Ren G. Huang Y. Z. Wei T. B. Liu J. X. Lan X. Y. Lei C. Z.. (2014). Linkage disequilibrium and haplotype distribution of the bovine LHX4 gene in relation to growth. Gene 538, 354–360. 10.1016/j.gene.2013.12.037 24463020

[B29] Ren J. Duan Y. Qiao R. Yao F. Zhang Z. Yang B.. (2011). A missense mutation in PPARD causes a major QTL effect on ear size in pigs. PLoS Genet. 7:e1002043. 10.1371/journal.pgen.1002043 21573137 PMC3088719

[B30] Shaat I. Mäki-Tanila A. (2009). Variation in direct and maternal genetic effects for meat production traits in Egyptian Zaraibi goats. J. Anim. Breed. Genet. 126, 198–208. 10.1111/j.1439-0388.2008.00784.x 19646148

[B31] Sharma R. Ahlawat S. Maitra A. Roy M. Mandakmale S. Tantia M. S. (2013). Polymorphism of BMP4 gene in Indian goat breeds differing in prolifcacy. Gene 532, 140–145. 10.1016/j.gene.2013.08.086 24013084

[B32] Soldner F. Stelzer Y. Shivalila C. S. Abraham B. J. Latourelle J. C. Barrasa M. I.. (2016). Parkinson-associated risk variant in distal enhancer of α-synuclein modulates target gene expression. Nature 533, 95–99. 10.1038/nature17939 27096366 PMC5042324

[B33] Teperek M. Simeone A. Gaggioli V. Miyamoto K. Allen G. E. Erkek S.. (2016). Sperm is epigenetically programmed to regulate gene transcription in embryos. Genome Res. 26, 1034–1046. 10.1101/gr.201541.115 27034506 PMC4971762

[B34] Tomas N. Venkatachalapathy T. Aravindakshan T. Raghavan K. C. (2016). Molecular cloning SNP detection and association analysis of 5′ ?anking region of the goat IGF1 gene with prolifcacy. Anim. Reprod. Sci. 167, 8–15. 10.1016/j.anireprosci.2016.01.016 26852275

[B35] Vandesompele J. De Preter K. Pattyn F. Poppe B. Van Roy N. De Paepe A.. (2002). Accurate normalization of real-time quantitative RT-PCR data by geometric averaging of multiple internal control genes. Genome. Biol. 3:RESEARCH0034. 10.1186/gb-2002-3-7-research0034 12184808 PMC126239

[B36] Van Laere A. S. Nguyen M. Braunschweig M. Nezer C. Collette C. Moreau L.. (2003). A regulatory mutation in IGF2 causes a major QTL effect on muscle growth in the pig. Nature 425, 832–836. 10.1038/nature02064 14574411

[B37] Wang C. Zhang H. Niu L. Guo J. Jia X. Wang L.. (2015). The novel SNPs of leptin gene and their associations with growth traits in Chinese Nanjiang Yellow goat. Gene 572, 35–41. 10.1016/j.gene.2015.06.073 26142105

[B38] Wang X. Y. Yang Q. Wang K. Zhang S. H. Pan C. Y. Chen H.. (2017). A novel 12-bp indel polymorphism within the GDF9 gene is significantly associated with litter size and growth traits in goats. Anim. Genet. 48, 735–736. 10.1111/age.12617 29023802

[B39] Wu P. Yang Q. Wang K. Zhou J. Ma J. Tang Q. (2018). Single step genome-wide association studies based on genotyping by sequence data reveals novel loci for the litter traits of domestic pigs. Genomics 110, 171–179. 10.1016/j.ygeno.2017.09.009 28943389

[B40] Xu K. Chen X. Yang H. Xu Y. He Y. Wang C.. (2017). Maternal Sall4 Is indispensable for epigenetic maturation of mouse oocytes. J. Biol. Chem. 292, 1798–1807. 10.1074/jbc.M116.767061 28031467 PMC5290953

[B41] Yang Q. Yan H. Li J. Xu H. Wang K. Zhu H.. (2017). A novel 14-bp duplicated deletion within goat GHR gene is significantly associated with growth traits and litter size. Anim. Genet. 48, 499–500. 10.1111/age.12551 28295460

[B42] Yao X. Tang F. Yu M. Zhu H. Chu Z. Li M.. (2014). Expression profile of Nanos2 gene in dairy goat and its inhibitory effect on Stra8 during meiosis. Cell Prolif. 47, 396–405. 10.1111/cpr.12128 25195564 PMC6495812

[B43] Yap D. B. Walker D. C. Prentice L. M. McKinney S. Turashvili G. Mooslehner A. K.. (2011). Mll5 is required for normal spermatogenesis. PLoS ONE 6:e27127. 10.1371/journal.pone.0027127 22069496 PMC3206077

[B44] Yu S. Zhang P. Dong W. Zeng W. Pan C. (2017). Identification of stem leydig cells derived from pig testicular interstitium. Stem Cells Int. 2017:2740272. 10.1155/2017/2740272 28243257 PMC5294379

[B45] Zhan W. (2015). The Study of Spermatogenesis and Seminiferous Performance of Liaoning Cashmere Goat Ram. Master's thesis, Jilin Agricultural University, Changchun.

[B46] Zhang S. H. Xu H. Liu X. F. Yang Q. Pan C. Y. Lei C. Z.. (2017). The muscle development transcriptome landscape of ovariectomized goat. R. Soc. Open. Sci. 4:171415. 10.1098/rsos.171415 29308264 PMC5750031

[B47] Zhao H. Wu X. Cai H. F. Pan C. Y. Lei C. Z. Chen H.. (2013). Genetic variants and effects on milk traits of the caprine paired-like homeodomain transcription factor 2 (PITX2) gene in dairy goats. Gene 532, 203–210. 10.1016/j.gene.2013.09.062 24076438

