# Supplementary figures and images for: Insertion/Deletion Within the KDM6A Gene Is Significantly Associated With Litter Size in Goat
Source: Front Genet. 2018 Mar 20;9:91. doi: 10.3389/fgene.2018.00091 (PMC5869274; doi:10.3389/fgene.2018.00091)

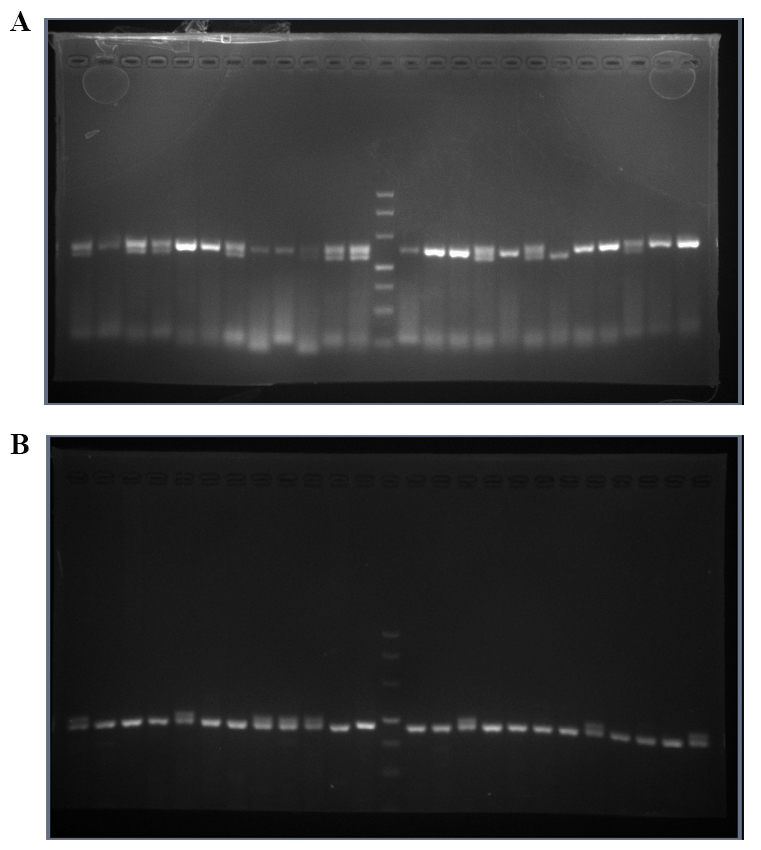

Supplement: Supplement Figure 1 — The original image of the electrophoresis diagrams. (A) 16 bp indel locus. (B) 5 bp indel locus. [file Image1.JPEG]
